# Long Noncoding RNAs in Interaction With RNA Binding Proteins in Hepatocellular Carcinoma

**DOI:** 10.5812/hepatmon.18794

**Published:** 2014-05-05

**Authors:** Ashraf Mohamadkhani

**Affiliations:** 1 Liver and Pancreatobiliary Disease Research Center, Digestive Disease Research institute, Shartati Hospital, Tehran University of Medical Sciences, Tehran, IR Iran

**Keywords:** Carcinoma, Hepatocellular, Long Noncoding RNA, RNA-Binding Proteins

## Abstract

**Background:**

Gene expression microarrays' analyses provide a description of long noncoding RNAs (lncRNAs) with lack of coding protein function that is often important in human cancer.

**Objectives:**

A number of lncRNAs that have been well characterized in hepatocellular carcinoma (HCC) have been scheduled in this study to discuss for protein–lncRNA interaction.

**Materials and Methods:**

The identified lncRNAs were analyzed by bioinformatics tools, starBase and lncRNA db, to anticipate the RNA-binding proteins (RBPs) that tend to interact to HCC-related lncRNAs. The most important predicted RBPs in interaction with well-known lncRNAs in HCC were briefly discussed.

**Results:**

The lncRNAs HOTTIP, H19, HOTAIR, MALAT1, antisense Igf2r (AIR), HOXA13, GTL2 (also called MEG3) and uc002mb have been reported in association with HCC. Besides, this study predicted that eIF4AIII, PTB and FUS were the most involved RBPs in interaction with HCC-related lncRNAs.

**Conclusions:**

This information provides an explanation for the previously valuable literature on the functions of lncRNAs and suggest for the novel therapeutic targeting.

## 1. Background

Progresses in whole genome expression profiling and transcriptome sequencing have been revealed new type of RNAs and their specific functions in eukaryotic cells (1). Long non-coding RNAs (lncRNAs) comprise the mainstream of transcripts that are larger than 200 nucleotides (nt) and not translated into proteins. Application of next generation sequencing and high-resolution of microarray techniques discovered more than 14,000 lncRNA transcripts in five classes of sense, antisense, bidirectional, intronic, and intergenic (2). The majority of mammalian coding genes have complementary noncoding antisense transcription (3). Many of which that are implicated in a cis-acting performance, mediate the modifications of native chromatin and the expression of neighboring genes in gene silencing way (3). Conformational changes in essential domains of lncRNAs will capable them to interact to the complementary base pair of other RNAs as well as proteins and perhaps DNA. Alternative splicing of RNAs are responsible for constructing domains in lncRNAs structures (1, 4). Recently, many novel approaches have been developed to identify the functional role of lncRNAs molecules in the development of human diseases. These molecules regulate many biological processes such as epigenetic modulators that potentially alter the levels of mRNA transcripts in human cancer (5). Application of lncRNAs as cancer diagnostic or prognostic biomarkers has been reported in several studies (6). For example, PCA3, a well-known prostate-specific lncRNA, is markedly overexpressed in prostate cancer and has been offered as cancer diagnostic biomarker (4). Previous studies also showed that patients with higher lncRNAs expression had poorer prognosis in liver cancer (7). Expression of highly upregulated in liver cancer (HULC) lncRNA in plasma has been known as a novel mRNA-like lncRNA biomarker for the diagnosis of HCC (8).

Recent study showed that lncRNAs are associated with cancer subtypes or may have a tumor-promoting or suppressing function (5). Genetic studies have proved that a significant number of non-coding RNAs (ncRNAs) are associated with hepatocellular carcinoma (HCC) as the most common types of cancer in areas where chronic viral hepatitis are prevalent (9). Essentially, lncRNAs are involved in the pathogenesis of HCC through regulation of carcinoma-related signaling pathways such as MAPK signaling pathway (10). The etiology and the overwhelming majority of HCC cases are associated with a chronic inflammatory process in viral hepatitis. Both hepatitis B virus (HBV) DNA and HCV RNA could change the cellular regulatory mechanisms and leading to HCC (11, 12). It has been found that chromosomal instability, cellular gene expression alteration and in particular the transactivating genes by HBx are important reasons in pathogenesis of HBV-associated HCC (13, 14). Generally, there are specific motifs within the binding sites of lncRNAs for protein regulatory factors (15, 16). Comprehensive RNA-binding proteins (RBP) dataset from various cell types have been provided by high-throughput CLIP-Seq technology. Additionally, several computational approaches have been developed to recognize lncRNAs-proteins interactions and help to find out the regulatory mechanisms in human cancer (17, 18). These methods highlight the potential uses of ncRNAs in early detection, diagnosis and therapy of cancers such as HCC.

## 2. Objectives

In this study HCC-related lncRNAs were extracted from literature to evaluate their possible interactions with RBPs by performing computational programs. Prediction of lncRNAs–RBPs interactions will be potentially useful to explore the molecular mechanisms that are regulated by lncRNAs and influence on the function of proteins in HCC. 

## 3. Materials and Methods

### 3.1. Search Strategy and Selection of Long non-Coding RNAs (LncRNAs)

PubMed and reference lists of relevant review articles were searched to retrieve HCC-related lncRNAs. All searches were updated in December 2013. Searching results were listed in Table 1 ; as shown, most of lncRNAs are overexpressed, which indicates an oncogenes-like role of lncRNAs in cancer biology.

**Table 1.** Identified lncRNAs in HCC From Definite Studies

LncRNAs	Gene Description	Functional Annotation	Reference
**HOTTIP**	HOXA is bidirectional transcript with HOXA13 (Homeobox protein Hox-A13)	Controls activation of several 5’ HOXA genes	(7, 10)
**H19**	Imprinted maternally expressed transcript at the Igf2 locus	Possibly will act as a tumor suppressor, influences growth via control of Igf2 expression.	(19-24)
**HOTAIR**	Hox antisense intergenic RNA, that is co-expressed with HOXC genes.	HOTAIR acts as a scaffold for protein complexes. Involve in chromatin modifications.	(25-27)
**HULC**	Highly Up-regulated in Liver Cancer, 500 bp in two exon polyadenylated transcript	Complicated in HBx-mediated hepatocarcinogenesis, downregulates the expression of some microRNAs, including miR-372	(28)
**MALAT1**	Metastasis-associated lung adenocarcinoma transcript 1	Highly conserved transcript that regulates the expression of metastasis-associated genes	(10, 29)
**antisense Igf2r (AIR)**	Transcribed from an antisense promoter located in intron 2 of the Igf2r (insulin-like growth-factor type-2 receptor) gene	Regulates genomic imprinting of a cluster of autosomal genes (Igf2r, Slc22a2 and Slc22a3) in cis	(30)
**GTL2 (also called MEG3)**	Gene-trap locus 2 (maternally expressed imprinted gene 3), had two major mRNA forms	Interacts with the tumor suppressor p53, and regulates p53 target gene expression	(30)

### 3.2. Prediction of RBP Binding Sites on LncRNA Target

StarBase v 2.0 (http://starbase.sysu.edu.cn/) has distinctive features and improved as Database, Visualizer, and Analyzer. This server was used to discover the lncRNAs- RBPs interactions. Included lncRNAs from published studies was administered to identify the possible RBP binding sites among them.

### 3.3. RNA-Binding Protein (RBP) Function Information

StarBase has provided promising information of predicted RBPs for each lncRNA target. This server provides functional insights on target sites within RBP-lncRNA interaction, shows the target location on chromosomes and the number of transcripts by Ensembl ID. LncRNAdb (http://www.lncrnadb.org/) is a valuable database to provide information of annotated lncRNAs including sequences, structural information, genomic context, expression, subcellular localization, conservation and functional evidence. The summarized information of lncRNAs and related RBPs were indicated in Table 2.

**Table 2.** The Summarized Information of lncRNAs and Related RBPs

LncRNAgene symbol	RNA-binding proteins (RBP)	Target Location	Target Transcripts (Ensembl IDs)
**HOTTIP**	PTB- eIF4AIII- DGCR8- FUS- UPF1	Chromosome 7	ENST00000472494 ENST00000605136
	interaction with the WDR5 / MLL complex		ENST00000521028 ENST00000472494
**H19**	PTB, eIF4AIII, DGCR8, FUS	Chromosome 11	ENST00000414790 ENST00000412788 ENST00000439725 ENST00000411861 ENST00000442037 ENST00000422826 ENST00000446406 ENST00000417089 ENST00000431095 ENST00000411754
**uc002mb**	Un-predictable		
**HOTAIR**	HuR, IGF2BP1, IGF2BP2, IGF2BP3, PUM2, eIF4AIII, FMRP, FUS, LIN28A, LIN28B, C22ORF28, ZC3H7B, U2AF65, hnRNPC,	Chromosome 12	ENST00000424518 ENST00000455246 ENST00000425595 ENST00000453875 ENST00000439545
	UPF1, HuR		
**MALAT1**	PTB, IGF2BP1, IGF2BP2, IGF2BP3, PUM2, QKI, TNRC6, eIF4AIII, DGCR8, FMRP, FUS, LIN28A, LIN28B, LIN28, MOV10, ALKBH5, C17ORF85,C22ORF28, CAPRIN1, ZC3H7B	Chromosome 11	ENST00000534336 ENST00000544868 ENST00000508832
**antisense Igf2r (AIR)**	Un-predictable		
**GTL2 (also called MEG3)**	PTB, eIF4AIII, DGCR8, FUS	Chromosome 14	ENST00000429159 ENST00000451743 ENST00000556736 ENST00000521404 ENST00000423456 ENST00000398518 ENST00000554639 ENST00000519709 ENST00000412736 ENST00000520714 ENST00000522771 ENST00000424076 ENST00000452120 ENST00000523671

## 4. Results 

Experimental data showed the achievement of lncRNAs in regulation of gene expression and most often, they are poor prognostic biomarker in patients with hepatocellular carcinoma (HCC) (8, 25, 26). lncRNA expression is significantly more cell type–specific, Table 1 represents the list of candidate driver lncRNAs in HCC from selected studies. These studies exhibit the expression of lncRNAs HOTTIP, H19, HOTAIR, MALAT1, antisense Igf2r (AIR), HOXA13, GTL2 (also called MEG3) and uc002 mb in HCC. To be aware of the molecular mechanisms of lncRNAs in HCC, it was assumed that lncRNAs are produced to regulate gene expression through their interaction with RBPs, therefore, the binding proteins to lncRNAs needed to be identified. Notably, RNA-binding proteins are the key factors in the regulation of gene expression (15, 16).

The next generation sequencing techniques, RNA immunoprecipitation and deletion mapping, quantitative real-time PCR and DNA microarray were the used methods in literature for the identification and potential function assessment of cancer-related lncRNAs. However, predicting RNA target proteins has been a topic of active research. Calculating the sequence similarity average between transcripts and their putative target protein sequences are the base of predicting tools. StarBase is the first database that provides the RNA-protein interaction networks to identify the most popular RBPs. Table 2 revealed predicted human RNA-binding proteins that interact with HCC-related lncRNAs. H19 was one of the first known and most studied lncRNAs, which has indispensable role for regulating of development and disease disorders (19-21). The regulatory function of H19 on the growth through the control of insulin-like growth factor II (IGF2) expression were evaluated by Ripoche et al. that showed the overgrowth phenotype in H19 silencing cells (31). IGF2 and H19 are two imprinted genes in humans with expression of the maternal H19 and paternal IGF2 alleles that are neighboring positioned at chromosome 11p15.5 (32). High expression of lncRNA H19 and its association with cell growth in the development of HCC has been well documented (20). However the biological function and regulatory mechanism of this conserved RNA remain largely unknown. H19 organized a complex of PCAF with acetyltransferase function and RNAPol II to activate miR-200 for higher acetylation of histones as important process in transcription (21). However, we anticipated that H19 interact with PTB, eIF4AIII, DGCR8 and FUS regulatory binding proteins (Table 2). The lncRNA HULC, promoted cell proliferation and involved in HBx-mediated hepatocarcinogenesis by the use of cAMP-responsive element-binding protein (28). However there were no anticipated RBPs for HULC in the databases. The lncRNA HOTAIR is a new potential marker of HCC (15, 25, 26). Analysis of microarray gene expression data showed the increased level of HOTAIR in HBV related HCC compare to normal liver tissues. Microarray expression analysis showed that high expression level of HOTAIR was an independent predictive for shorter recurrence-free survival of HCC after liver transplantation (27). Indeed, the marked up-regulation of lncRNAs MALAT1 and HOTTIP in HCV and HBV-associated HCC have been described (33, 34). MALAT1 with 8.7-kb in length has been associated with metastasis and poor prognosis of HCC according to histology findings. It is famous as an oncogene, because it regulates alternative splicing of endogenous target genes that are involved in cancer (29). HOTTIP (the transcript of HOXA at the distal tip with 3.8 kb) directly controls the HOXA locus gene expression via interaction with the complex of transcription factors WDR5 and MLL histone methyltransferase protein. Hox genes have regulatory functions to control the timing and route of development. Deregulation of Hox genes are involved in hepatocarcinogenesis (7, 35). Notably, researchers have demonstrated that down regulation of HOTAIR , MALAT1 and HOTTIP can reduce cell viability and cell invasion in tumoral cells, but rise TNF-α induced apoptosis (10, 27, 29). This study anticipated that eIF4AIII, PTB and FUS were the most implicated RBPs in interaction with HCC related lncRNAs. Eukaryotic initiation factor 4A-III (eIF4AIII) is a member of Define DEAD box proteins, a group of proteins that are implicated in regulation of a range of metabolic processes through interaction with RNA or other nucleic acids. Indeed polypyrimidine tract-binding protein, PTB or hnRNPI is known as a splicing regulator with functional role in mRNA stability and localization. Also RNA-binding protein FUS (fused in sarcoma) is unique and famous for protein regulatory function in transcription and DNA repair activity.

## 5. Discussion 

It has been well documented that at least 90 % of the human genome is actively transcribed. Currently, it has been documented that non-protein coding transcripts have regulatory function in cancer biology mainly through their relationships with RNA-binding proteins (RBP) in a sequence-specific manner (7, 15, 25, 36). 

These data demonstrated that the interaction of lncRNAs with RBPs might effect on the epigenetic regulatory function of lncRNAs molecules in mRNA inactivation and therefore, influence in common cellular functions. Furthermore, RBPs interacting with mRNA for biological function are probably blocked in lncRNAs-RBPs interactions. These finding suggest for oncogenic role of HCC-related lncRNAs. 

Analyses of human genome have revealed prevalent large RNA transcripts that are long non-coding RNAs (lncRNAs). These molecules have regulatory impact on gene expression in interaction with human genome and RNAs. LncRNAs are also organized in close relationship with RBPs regulatory proteins that suggest the importance of regulatory binding proteins in biological role of lncRNAs. The present study has been focused on well-defined lncRNAs in HCC and their interaction with regulatory proteins. H19, HOTAIR, MALAT1 and HOTTIP are most common lncRNAs in HCC. These lncRNAs are essential in many biological events for instance cell-proliferation and differentiation, apoptosis and tumorigenesis via their impact on RBPs. A few approaches are available for predicting interactions between lncRNAs and proteins. Here, the StarBase database showed that eIF4AIII, PTB and FUS were the most implicated RBPs in interaction with HCC related lncRNAs. Engagements of these RBPs by lncRNAs probably explain the tumorigenicity in HCC and opens new opportunities for novel therapeutic targets in HCC.
